# An examining the static and dynamic mechanical characteristics of milled ramie root reinforced polyester composites

**DOI:** 10.1038/s41598-023-44088-5

**Published:** 2023-10-10

**Authors:** T. VarunKumar, M. Jayaraj, N. Nagaprasad, Jule Leta Tesfaye, R. Shanmugam, Ramaswamy Krishnaraj

**Affiliations:** 1 Department of Mechanical Engineering, P.A College of Engineering and Technology, Pollachi, Tamilnadu 642002 India; 2 Department of Mechanical Engineering, Dr.Mahalingam College of Engineering and Technology, Pollachi, Tamilnadu 642003 India; 3 Department of Mechanical Engineering, ULTRA College of Engineering and Technology, Madurai, Tamilnadu 625 104 India; 4 College of Natural and Computational Science, Department of Physics, Dambi Dollo University, Dambi Dollo, Ethiopia; 5 Centre for Excellence in Technology Transfer and Incubation, Dambi Dollo University, Dambi Dollo, Ethiopia; 6 TIFAC, CORE-HD, Department of Pharmacognosy, JSS College of Pharmacy, JSS Academy of Higher Education and Research, Ooty, Nilgiris, Tamil Nadu India; 7 Department of Mechanical Engineering, Dambi Dollo University, Dambi Dollo, Ethiopia

**Keywords:** Energy science and technology, Engineering, Materials science, Physics

## Abstract

This research works discuss about the effective utilization of waste Ramie Root, that has been in reinforced polyester composites, powdered fillers that have not been treated are used. Four different composites plate were formed with compression moulding technique process consisting of 20, 30 and 40% of Powdered Ramie Root with 80, 70 and 60% unsaturated polyester resin, respectively. The maximum mechanical properties were observed for the composite with 30:70 weight volume percentages of milled ramie root synthetic reinforced polyester. The findings show that the glass transition temperature, storage modulus, and loss factors all rise when the composition of composites changes. Additionally, the powder cohesion force (bonding strength) has a greater impact on dynamic mechanical properties. Thermo-gravimetric the inclusion of Ramie Root powder caused the thermal deterioration peak of the composite to move from 370 °C to 418 °C, according to analysis (TGA) conducted under flowing oxygen. According to the measurement of water absorption, the ideal weight ratio of Fiber: Unsaturated Polyester Resin is 30:70, which modifies the fibres’ surfaces and ensures optimal adhesion between the fibre and matrix in composite materials. Scanning electron microscopic investigation is done to ascertain the fracture behaviour of the composite. As a result of their stability, high tensile strength, and bending stiffness, the produced composites can be used in light-load applications by material technologists.

## Introduction

Polyester-reinforced composites containing synthetic fibres, i.e., (glass and aramid), have boosted several qualities like toughness, stiffness, and superior strength-to-weight ratio as compared to the addition of fillers, such as wood and various types of fillers alone^1–3^. Some researchers and business sectors are interested in the mechanical strength of polyester reinforced with natural fibres.

Numerous studies have been conducted on polyester reinforced with coir fibres, rice husk, jute fibres, seashells, straw, and other Additionally demonstrated to be a crucial reinforcement in both types of polymers^4–8^. Based on natural filler reinforcement, the impact a waste filler's surface treatment and its still need further attention for lowering natural fiber's water absorption capabilities. Hybrid epoxy composites' mechanical and water-absorbing qualities based on jute and banana fibres were evaluated. The 50/50 weight ratio composites were thought to have the best mechanical and absorption resistance properties. The jute and banana fibres were treated with fillers, has improved their mechanical qualities significantly. As a result, the inclusion of fillers improved the strength-to-weight ratio^9^. We examined the thermomechanical characteristics of epoxy composites. to be better, when the alkali treated fillers were utilised in comparison with the untreated powder, Epoxy composites were tested utilising reinforced waste peanut shell powder^10^. Using the created polypropylene composites based on discarded shellfish shell bio-filler and calcium carbonate filler, it was shown that adding shellfish shell bio-filler to polypropylene boosted its mechanical properties when compared to adding calcium carbonate^11^. Studies demonstrate how different filler amounts and fibre lengths affect the mechanical properties of calcium carbonate-filled coir fibre composites. The results exhibits that the required properties will increase with the optimal fibre length and filler contents^4^. Studied the impact of the reinforced natural rubber/epoxy fabrics made of jute, sisal, and E-glass composite's mechanical and tribological characteristics and it was found that the filler loading boosted the mechanical characteristics and lowered the rate of wear when it was 10 wt%, which was the best one^12^ Chopped glass fibre epoxy. The creation of SiC-loaded composites and chopped glass fibre epoxy composites. The findings indicate that tensile and flexural properties are improved by a 10-weight percent addition of SiC, while impact and hardness qualities are improved by a 15-weight percent addition of SiC^13^. Jute epoxy composites were filled Using reinforced Azadirachta indica seed powder and wasted Camellia sinensis powder, the researchers discovered that adding 10 weight percent of the azadirachta seed powder improved the composites' mechanical capabilities. 10 weight percent of the spent Camellia sinensis fillers increased the material's thermal stability^14^. When calatropsis gigantea Jute-epoxy composites that are powder-filled thermo-mechanical behaviour was looked at, it was discovered that adding 10 weight percent of filler-based composites increased the materials' mechanical and thermal stability^15^. examined the jute fibre and epoxy composite's mechanical characteristics after being filled with granite powder. When the filler percentage grew by more than 10 weight percent, it was discovered that the composite's ultimate tensile strength and ultimate flexural strength had reduced^16^.

From the research gap, found that no one has developed the Milled Ramie Root composite material. Investigation of static and dynamic mechanical properties results are enough for the sustainability of novel fiber polymer. Based on the current research, only limited studies were carried out on the composites were developed by reinforcing Ramie Root powder with 20, 30 and 40 wt%. Here, The developed composites' mechanical characteristics, such as their tensile, flexural, impact, and dynamic mechanical properties, such as their storage modulus, loss modulus, and damping factor, were characterized. Additionally, water absorption and thermal stability analysis were made using thermo gravimetric analysis (TGA). The tested composites were examined using scanner electron microscopy (SEM) to discover the optimum uses.

## Materials and methods

### Materials

**Figure 1:**
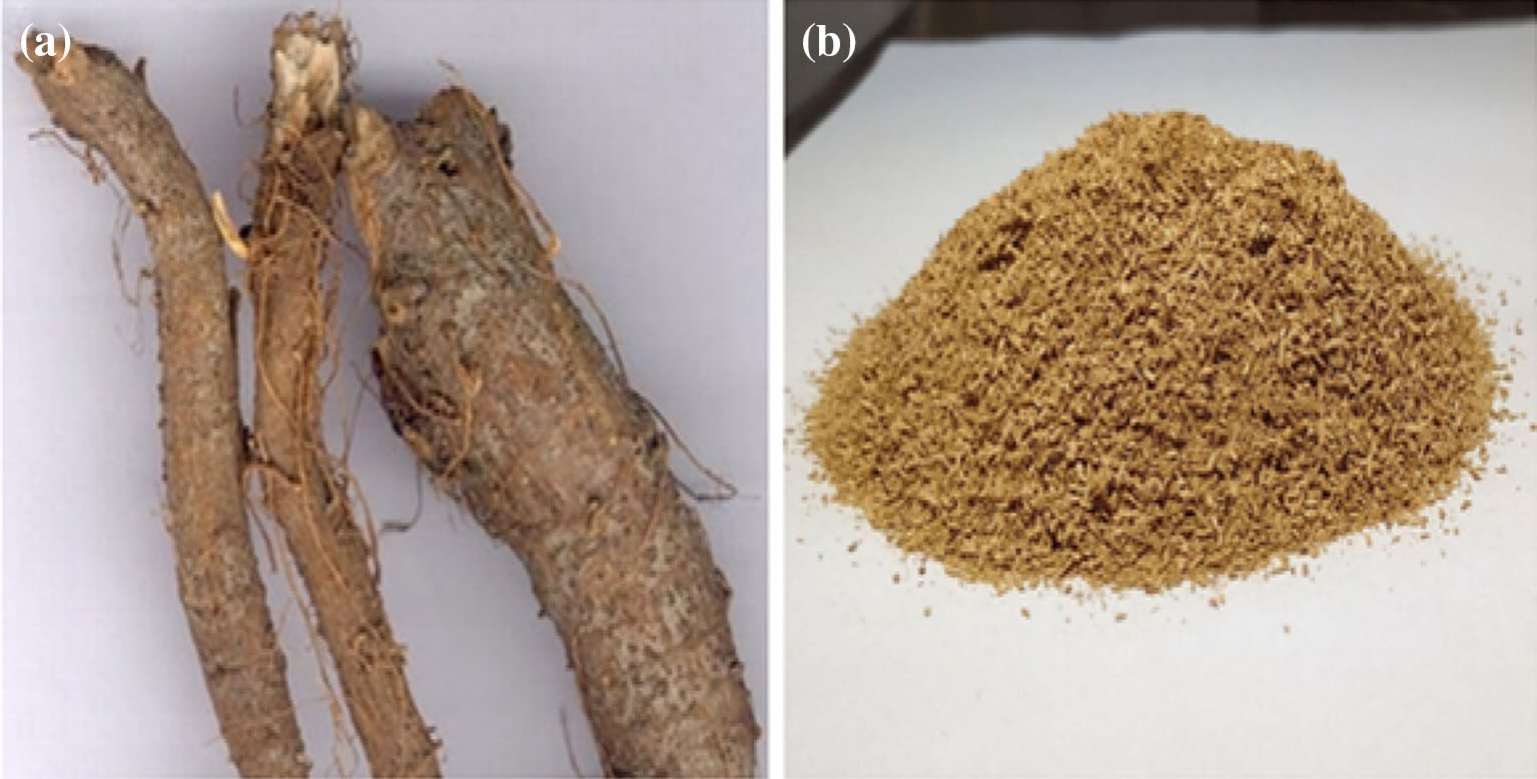
(**a**) Ramie root (**b**) Ramie root powder.

Ramie roots were extracted from the deeproots of ramie plant (Boehmeria nivea) and ramie roots were milled from the milling machine for producing the composite materials. The ramie roots and milled powder are shown in Fig. 1. The plant we have used in this report was cultivated in the local area of Pollachi, Tamilnadu, India. This study complies with relevant international, national, institutional and legislative guidelines^17^. To Collect the plant Permissions were obtained P.A. College of Engineering, Research department, Pollachi, Coimbatore-642 002, Tamilnadu, India.

### Fabrication of composite

**Figure 2:**
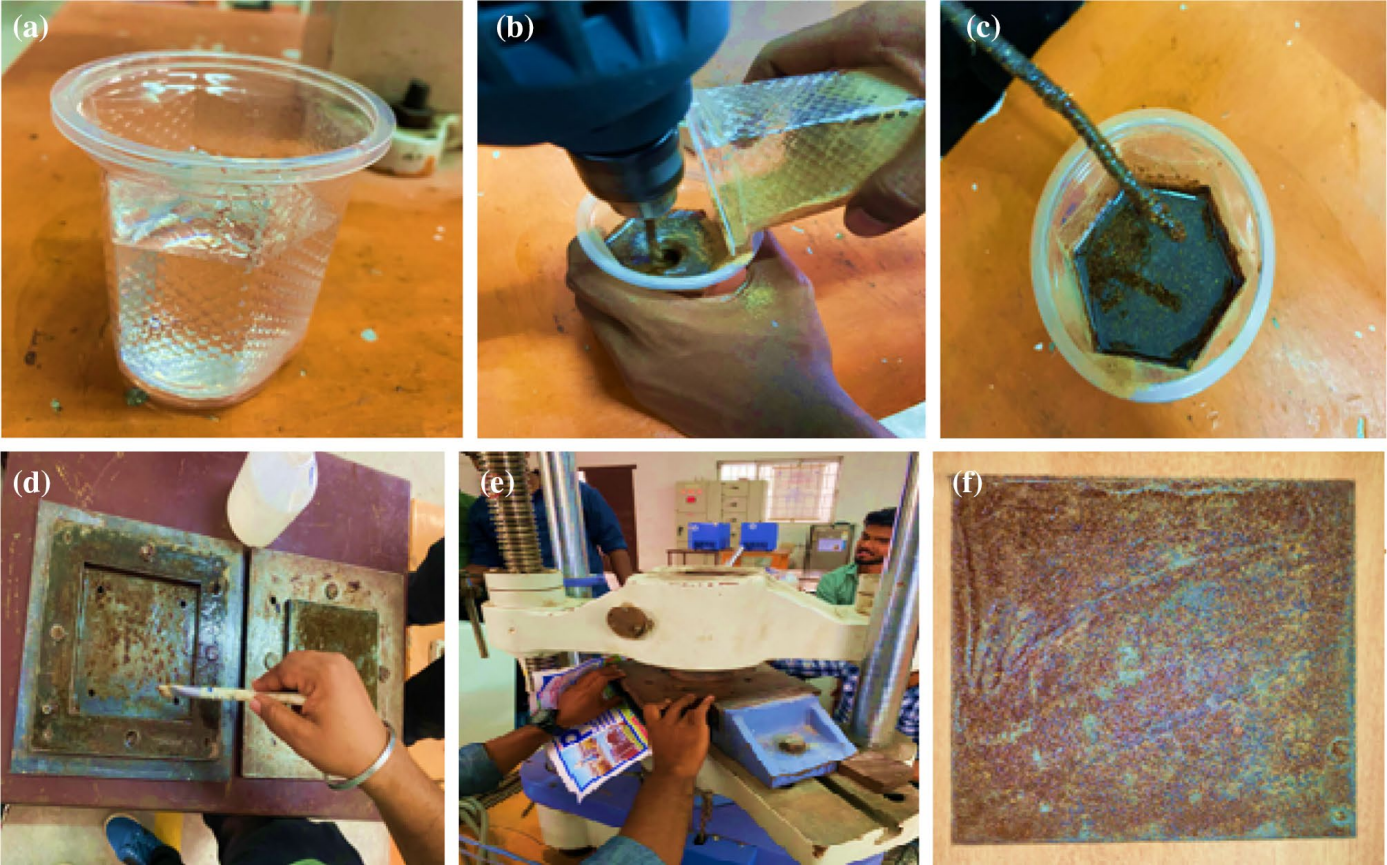
Fabrication process of milled ramie root reinforced polyester composites ^21^. (**a**) Resin (**b**) Mixing of resin and fibre (**c**) Die cleaning (**d**) Compression (**e**) Remove the loading (**f**) Composite materials.

To create the natural composites, Ramie Root powder was bonded with unsaturated polyester resin. The hardener accelerator was subsequently blended by volume with the unsaturated polyester resin in a 10:1 ratio, as advised. To dissolve the resin and the hardener The mixture was manually stirred in the matrix. The composite slabs were made using traditional hand lay-up techniques, which were followed by light compression moulding techniques^18,19^. A stainless steel mould with the following measurements: 150 mm 150 mm 3 mm was employed^19,20^. A releasing agent was added to make it simple to remove the composite from the mould once it had dried^18,21^. Before The cast of each composite was taken out of the mould and cured for 24 h under a weight of 50 kg^19,22^. The following composite plates (NT, R20C, R30C, & R40C) were created using plain resin, 20%, 30%, and 40% Ramie Root powdered with 80, 70, and 60% unsaturated polyester. The designation of powdered Ramie Root reinforced polyester composite is shown in Table 1. Figure 2 depicts the production of Ramie Root powdered reinforced composite.

**Table 1:** Designation of powdered ramie root reinforced polyester composite.

S.No	Name of the composite	Composite designation
1	Neat resin	NT
2	20% of powdered ramie root with 80% unsaturated polyester composite	R20C
3	30% of powdered ramie root with 70% unsaturated polyester composite	R30C
4	40% of powdered ramie root with 60% unsaturated polyester composite	R40C

### Mechanical static test

**Figure 3:**
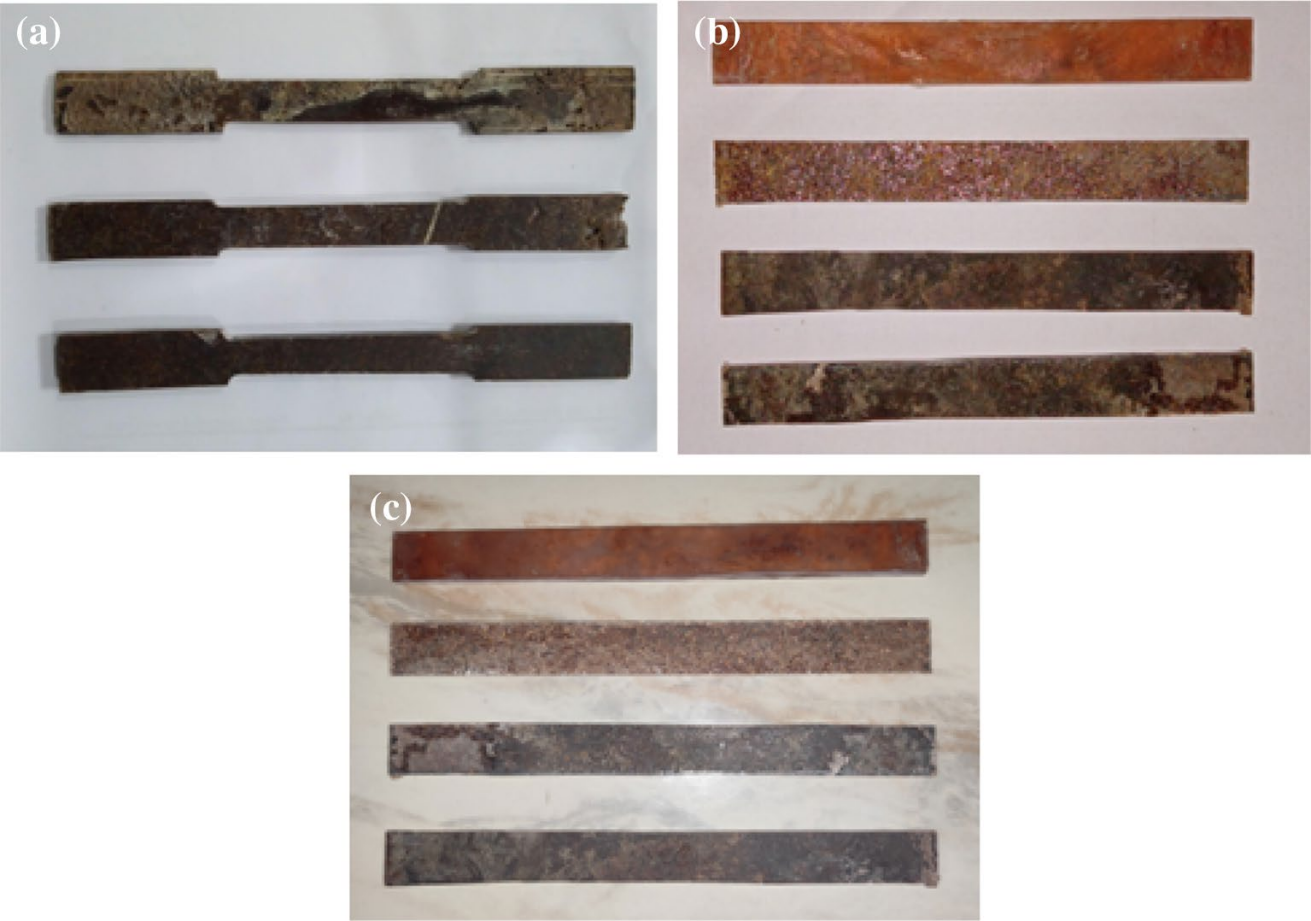
(**a**) Tensile (**b**) Flexural and (**c**) Impact specimen for powdered ramie root reinforced polyester composite.

To determine the tensile properties of the composites, an Instron tensile tester was used. The tensile characteristics of the composites were tested using the ASTM: D 638 standard at a crosshead speed of 5 mm/min^23^. The flexural strength of the specimens was tested in accordance with ASTM D790-03 using a Kalpak Universal Testing Machine with a 20kN capacity and a crosshead speed of 2 mm per minute. For the impact test in this instance, specimens of the composite were cut out in line with ASTM: D256^24–26^. For each test, three samples were examined, and the average outcomes were noted. The tensile, flexural, and impact. specimens are shown in Fig. 3.

### Test for water absorption

The water absorption behaviour of a powdered Ramie Root reinforced polyester composite was examined in accordance with ASTM D 570-99^27^. The specimens were submerged in water at room temperature to evaluate the kinetics of water absorption. Test samples were periodically taken out and immediately weighed. The quantity of water absorbed by the sample was calculated using an accurate 4-digit balance^27,28^.

Water absorption percentage is indicated by^21^

1 $$ {\text{Water}}\;{\text{soaking}}\;{\text{up}}\;{\text{W}}_{{\text{c}}} \left( \% \right) = {\text{W}}_{{2}} - {\text{W}}_{{1}} /{\text{W}}_{{2}} \times {1}00 $$

W1 is the weight (in grammes) before soaking in water, and W2 is the weight (in grammes) after soaking in water (g).

### Mechanical dynamic analysis

The visco elastic properties of the milling ramie root reinforced epoxy composite were examined by Seiko instruments DMA 6100 using the dynamic mechanical analyzer. Investigating the viscoelastic properties as a function of temperature involved a 3-point bending test. According to ASTM D 5023^15^, the composites were sliced into samples with diameters of 50, 13, and 3 mm. At a frequency of 1 Hz, experiments are run in the temperature range of 30 °C to 200 °C^28^ . The visco-elastic characteristics of the composite, including its storage modulus, loss modulus, and damping parameter, were examined.

### Thermo-gravimetric analysis (TGA) of composites

The composites' thermal consistency was assessed using the ASTM E 1131 standard. On a Perkin Elmer equipment, the specimen weight was analysed using TGA/DTG between 50 and 750 °C at a nitrogen atmosphere flow rate of 20 ml/min and a heating rate of 10 C/min^21^.

### Scanning electron microscopy (SEM)

An electron scanning microscope was utilised to study the Ramie Root composite samples after they underwent tensile fracture (Carl Zeiss EVO MA 15). In order to avoid electric charge while being examined, all specimens were coated with a extremely tiny coating of gold before being placed putting double-sided tape on an aluminium stub. Under typical secondary electron imaging circumstances, the SEM micrographs were created^20^.

## Result and discussions

### Tensile properties

**Figure 4:**
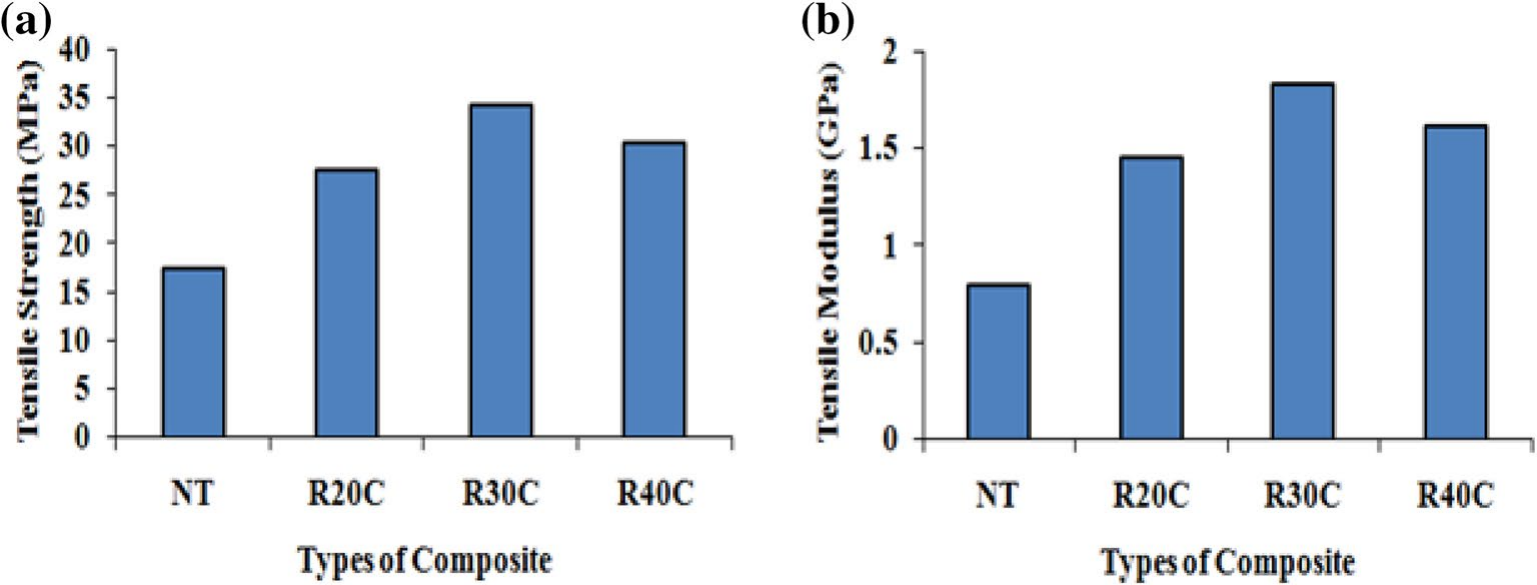
(**a**) Tensile strength (**b**) Tensile modulus of ramie root polyester composites.

Figure 4 indicates that the modulus and tensile strength of ramie root composites such as NT, R20C, R30C and R40C. The R30C composite exhibited 38.22, 50.32, and 43.91% increases in tensile strength and 41.37, 52.97, and 44.68% increases in tensile modulus compared with pure resin. The R30C composite reveal maximum tensile strength of 34.22 MPa and 1.83 GPa for the tensile modulus. Due to inadequate adhesion between the matrix and fibres, the tensile characteristics of R20C and R40C composites are diminished. Here, the appropriate weight fraction of ramie root powder causes powder segregation and also alters the crystallinity of the fibres by removing the fastening material, increasing the mechanical characteristics of R30C composites^20,21^.

### Adaptive propertiess

**Figure 5:**
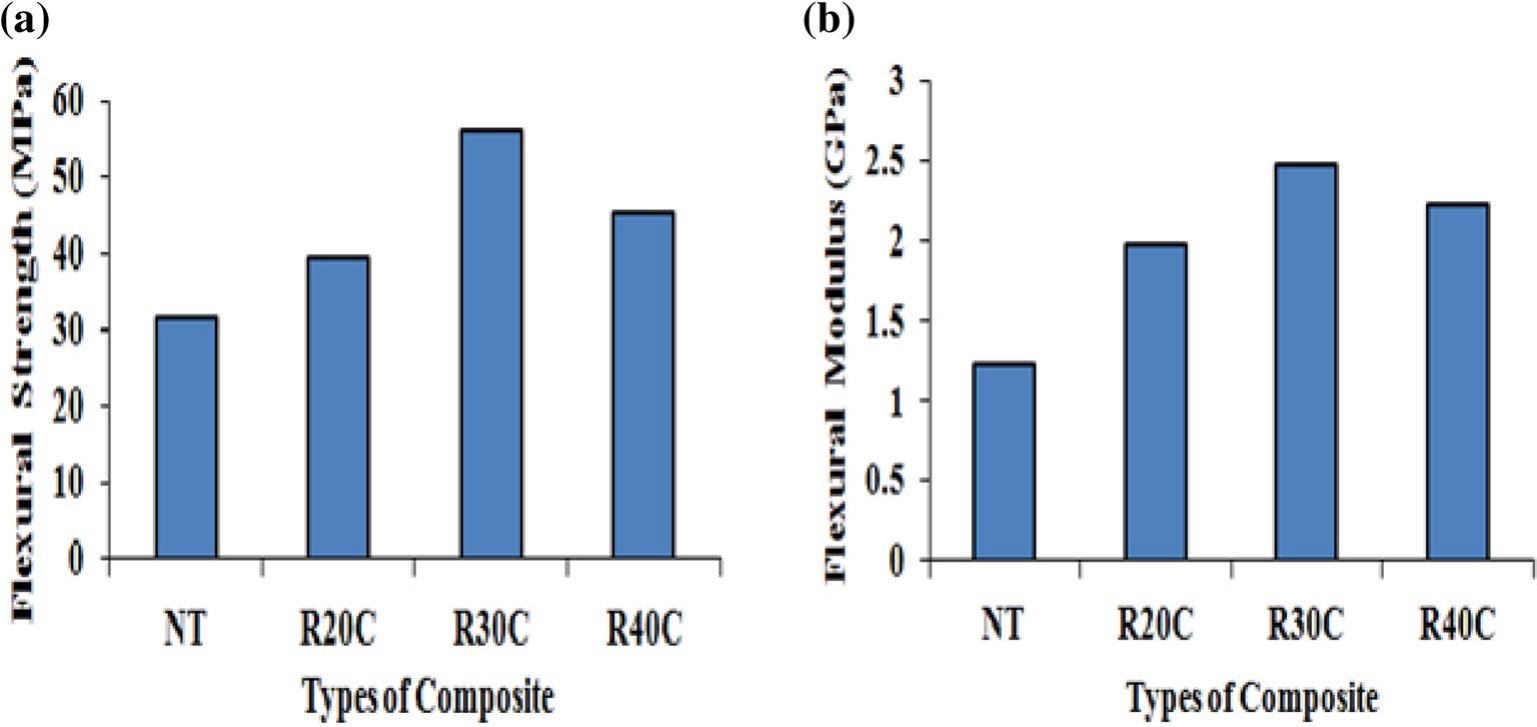
(**a**) Flexural strength (**b**) flexural modulus of ramie root polyester composites impact of properties

The modulus and flexural strength of ramie root composites such as NT, R20C, R30C and R40C shown in Fig. 5. The R30C composite showed 20.7, 44.02 and 30.9% increase in flexural strength and 38.5, 59.26 and 47.15% has increased compared to pure resin composites, in flexural modulus. Flexural strength of 56.03 MPa and a flexural modulus of 2.47 GPa are the maximum for Composite R30C. Because loads are delivered vertically on the composite's transverse axis, interlayer forces in powder-reinforced polyester matrix composites regulate the Flexural modulus and flexural strength. Hete, the composite's highest flexural properties show a strong connection between the fibre and matrix. In composites, the ideal weight percentage modifies the fiber's surface, which promotes strong adhesion between the powder and the substrate^18,21^.

**Figure 6:**
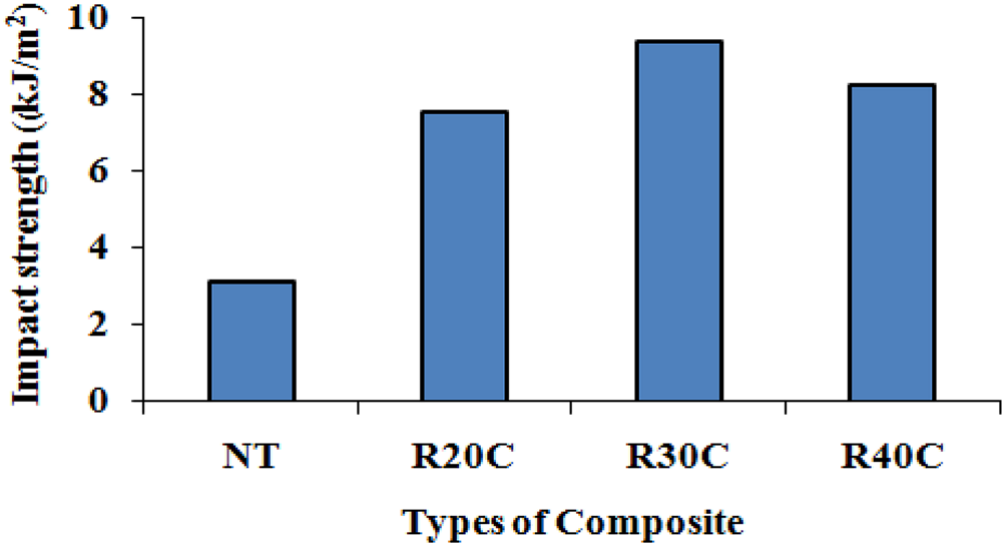
Impact strength and modulus of ramie root polyester composites.

Figure 6 displays the impact resistance of NT, R20C, R30C, and R40C composites. The outcomes demonstrate how the right powder weight ratio can increase composites' ability to withstand impacts. The binding strength between the fibre and the matrix directly affects the impact properties of composites. 9.36 kJ/m^2^ is the greatest impact strength recorded for Composite R30C. However, compared to neat resin, composite R30C has improved impact resistance by 57.29, 65.38, and 60.72%, respectively. During impact testing, the surface establishes a strong link between the matrix and the powder to absorb more energy as well as to stop the break from spreading ^18^. Table 2 show the Mechanical properties of Ramie Root fiber composite with different natural fiber composites^16^.

**Table 2:** Mechanical properties of ramie root fiber composite with different natural fiber composites.

Composite	Tensile strength (MPa)	Tensile modulus (GPa)	Flexural strength (MPa)	Flexural modulus (GPa)	Impact strength (kJ/m^2^)
Ramie*	3422	1.83	56.03	2.247	9.36
Jute	34.78	1.88	66.25	3.69	8.67
Straw	32.67	1.62	47.10	2.17	2.65
Sisal	28.55	1.49	53.42	4.25	9.61
Banana	17.69	1.03	33.51	1.59	9.36
Coir	18.61	1.16	31.15	1.50	3.91
Hemp	34.63	2.56	60.51	3.47	7.36
Kenaf	32.14	2.48	57.35	2.99	3.24

*Present work

### Water absorbing qualities

**Figure 7:**
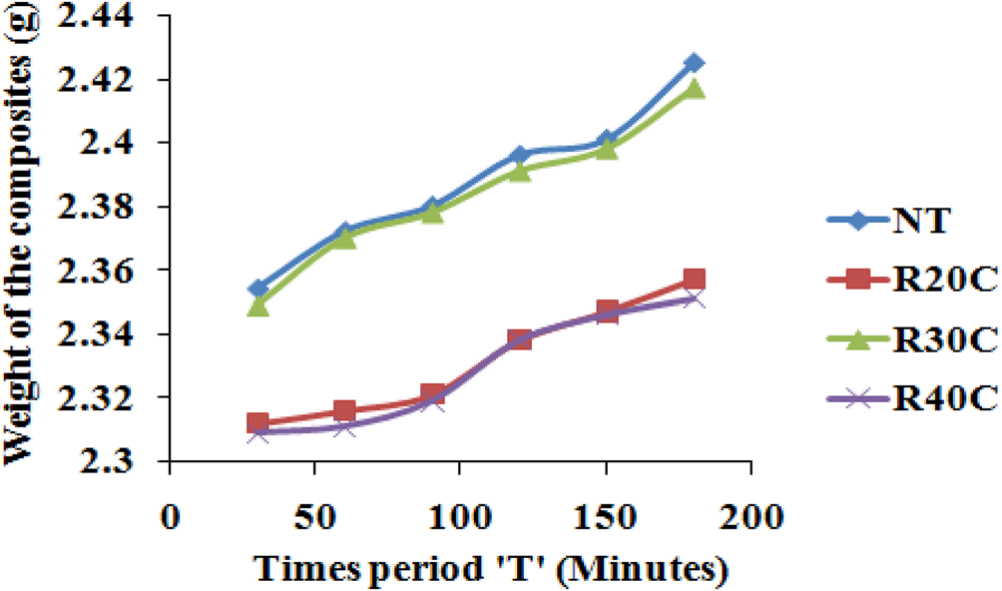
Water absorption weight of ramie root polyester composites.

The composites’ capacity to absorb water was evaluated during time intervals ranging from 30 to 180 min. Figure 7 shows the composite weight by hygroscopicity. Note that the amount of water absorbed by R20C is very small. Weight gain rate is determined using Eq. (1). For each deviation from the initial weight, the mixture weight increased significantly every 30 min. R40C exhibits the highest hygroscopicity. The fiber surface interactions are greater when the powder is high and the amount of pectin, lignin and hemicellulose, which are the major moisture-scavenging molecules of the powder is high. This explains the high hygroscopicity of the powder. As a result, treated fibres have comparatively low hygroscopicity as compared to untreated fibres^15,21^.

### Analysis using thermogravimetry

**Figure 8:**
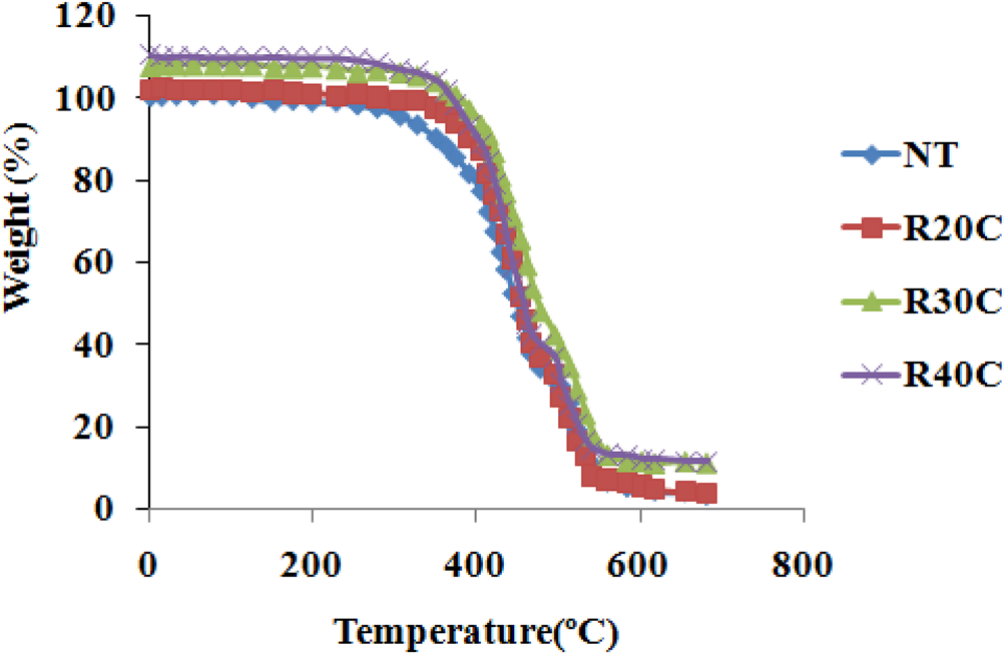
Thermo-gravimetric analysis curve oframie root polyester composites.

Figure 8 indicates the Ramie Root Polyester Composites Thermo-Gravimetric Analysis. This composite has been subjected to a single stage of thermal deterioration at temperatures between 30 °C and 600 °C. Additionally, in this test, the entire composite exhibited comparable breakdown behaviour. Lower temperatures facilitated the breakdown of leftover material of 10%, 12%, 16%, and 20% for NT, R20C, R30C, and R40C composites, respectively, from the four stages of decomposition. The initial step of degradation occurred in the composites at temperatures ranging from 170 °C to 200 °C. Moisture evaporation and solvent removal occurred in the composite. The second step of composite breakdown happened between 260 °C and 290 °C. This section was eliminated since it implied the degradation of substances such as lipids and waxes^19,27^. In the composite, the third step of degradation occurred between 340 °C and 400 °C. It has also occurred in the disintegration of hemicelluloses, lignin and cellulose, in addition to the degradation of soft segment and composite volatilization^28^. In the composites, the last step of breakdown occurred between 450 °C and 490 °C. The fibre and polyester composites are then degraded at this point. When compared to the raw resin, where the weight loss of all treated composites over 510 °C was found to be reduced^28^.

**Figure 9:**
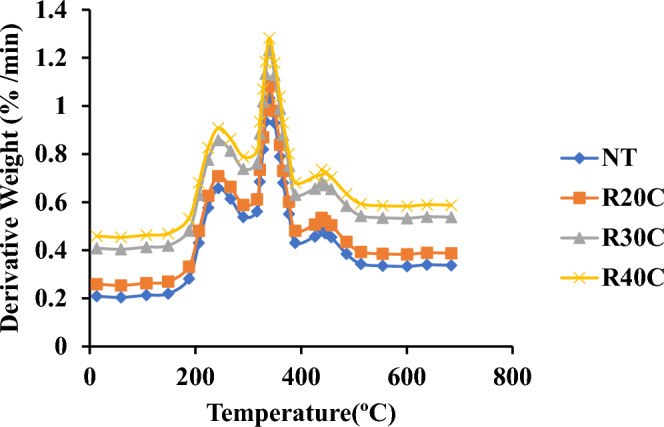
Differential thermal analysis of ramie root polyester composites.

Figure 9 displays the DTA curves for ramie root powdered polyester composites. The composite disintegrates as a result of endothermic and exothermic reactions occurring at various temperatures. In relation to the % weight growth in the relevant composite, weight loss occurs over NT, R20C, R30C, and R40C composites can withstand temperatures of 140 °C, 200 °C, 250 °C, and 590 °C, respectively. When plain resin is heated over 490 °C, a significant weight loss is seen.

### Mechanical dynamic analysis

#### Memory modulus (E′)

**Figure 10:**
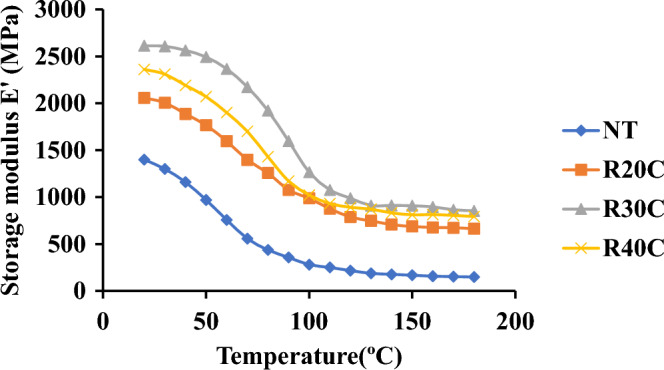
Storage modulus of ramie root polyester composites.

Ramie Root Polyester Composite's damping coefficient, storage modulus, and loss modulus. The findings demonstrate that the storage modulus (E′) values for R30C composites are at their highest. Additionally, it is seen that as temperature is steadily raised, the value of the storage modulus (E′) decreases. When compared to virgin plastic, non-hybrid reinforced composites almost always behave well as the temperature rises, as seen by the storage modulus (E′). In addition, the R30C composite achieved a large maximum storage value (E′) of 1312 MPa thanks to the fiber's improved impact on stress transfer crosslinking. The root powder has a coarse texture throughout the surface because interlocking between the reinforcement and the substrate increases cellulose content, which improves stress transfer^19^. Storage Modulus of Ramie Root Polyester Composites was depicted in Fig. 10.

#### Loss modulus (E″)

**Figure 11:**
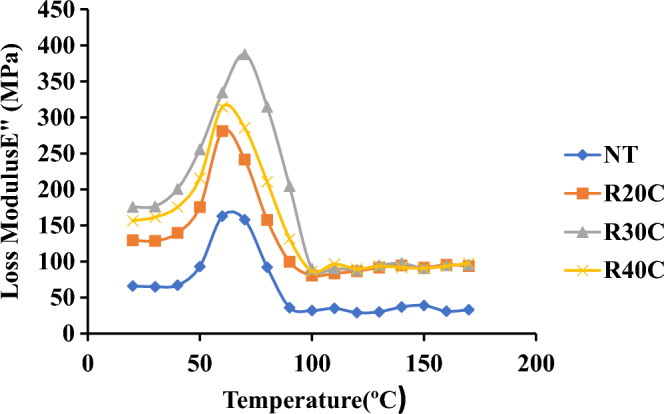
Loss modulus of ramie root polyester composite.

The NT, R20C, R30C and R40C showed peak values of 200.2 MPa, 214.56 MPa, 224.96 MPa and 231.4 MPa at 60 °C and 200 °C, respectively. Due to energy dissipation caused by thermal cycling under deformation carried out in viscoelastic materials, the highest loss modulus of R30C was measured^23^. Loss modulus plots show that hybrid composites containing ramie root powder lead to increased modulus peaks. Loss Modulus of Ramie Root Polyester Composite was illustrated in Fig. 11.

### Damping factor (Tan δ)

**Figure 12:**
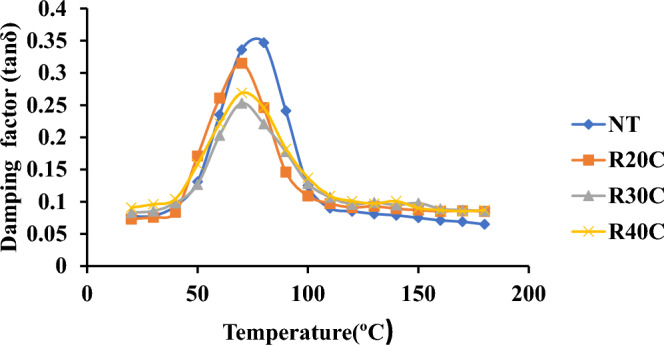
Damping factor of ramie root polyester composites.

The damping factor and composite material impact resistance can be related (Tan). R30C has a lower peak value of the damping factor, which is 0.174, because to its high cellulose content. indicating good surface bonding between the matrix and the powder. The damping factor value was observed to fluctuate greatly when compared to the hybrid composites. However, the weaker surface connection between the matrix and the powder is caused by the damping factor’s greater peak value. Damping Factor of Ramie Root Polyester Composites was shown in Fig. 12.

### Scanning electron microscopy

**Figure 13:**
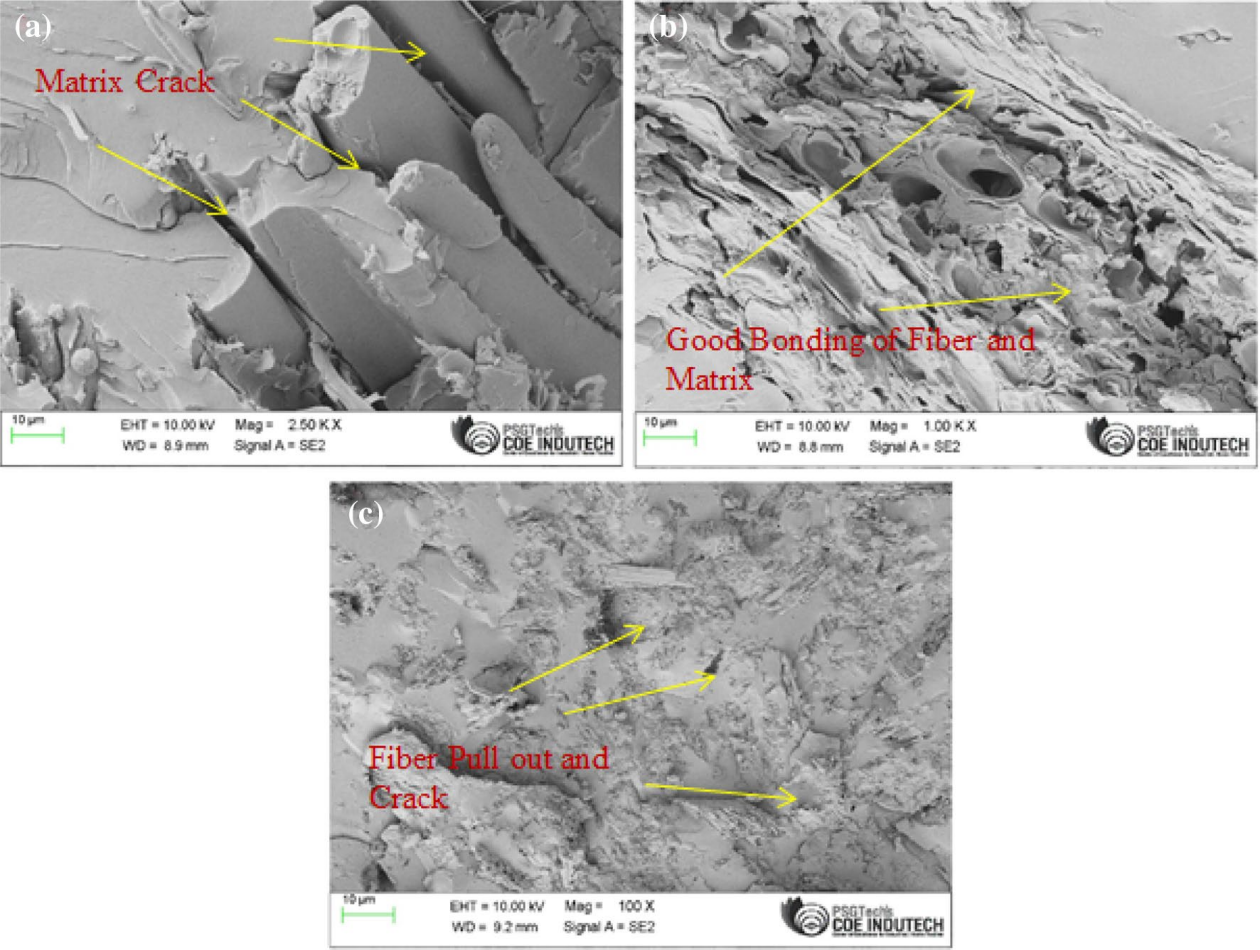
Scanning electron microscopy of (**a**) R20c (**b**) R30C and (**c**) R40C.

Reduced flexural strength is a result of proper mixing. Figure 13 displays a SEM image of appropriate mixing. Even if the fillers have filled the gaps in the composites for significant stress transmission, the protein layer does not attach to the matrix well in the case of composites, which causes filler debonding., as shown by the SEM images of R20C and R40C (Fig. 13a,c).The SEM picture shows a section of reinforced powder as well as the contact between the matrix and the reinforcement. The R30C composites (Fig. 13b) show a consistent distribution of fillers over the whole surface of the composite, except for the reinforcing surface. However, the interface of the matrix and the reinforcement, the fillers cover the minute gaps and eliminate fractures and holes, which impair the material's major qualities^18^. Therefore, The treated fillers that are present along the crack's route improve the filler's wetting and adherence to the matrix, or cross-linking, which causes the cracks to deflect in the composites and also pin themselves, as can be seen in the SEM picture of Fig. 13b.

## Conclusions

In this present study effort, powdered ramie root reinforced unsaturated polyester composites were generated by adopting the compression moulding process. Here, the conclusions that may be derived from the study's results are as follows: The R30C showed maximum 34.22 MPa in tensile strength, 56.03 MPa in flexural strength, and 9.36 kJ/m^2^ in impact strength. according to the randomly dispersed powdered ramie root reinforced unsaturated polyester composites. The SEM study also corroborated the findings of reduced pull-out and fracture. The boost in strength is attributable to the inclusion of the ramie root powder in the composites. The dynamic mechanical investigation of Powdered Ramie Root Reinforced Unsaturated Polyester Composites indicated that the R30C displayed the highest storage modulus(E′) of 1312 MPa and loss modulus(E″) of 231.4 Mpa and low damping element (tanδ) of 0.17. R20C was found to have significantly less water absorption than the other compositions, and R30C showed thermal degradation with a 20% residual mass between 500 °C and 600 °C. Since the mechanical and the moisture absorption are improved in the current composite, it may be applicable in regions where higher wear and water resistance are required such, doors, window panels, partition walls, wall roof, shoe sole and sound proof applications.

## Data Availability

The datasets used and analyzed during the current study are available from the corresponding author on request.
